# Comment on “Zoonotic and Non-zoonotic Parasites of Wild Rodents in Turkman Sahra, Northeastern Iran”

**Published:** 2018

**Authors:** Seyed Mahmoud SADJJADI

**Affiliations:** Dept. Parasitology and Mycology, School of Medicine, Shiraz University of Medical Sciences, Shiraz, Iran

## Dear Editor-in-Chief

I have read with great interest the article by Gholipoury et al., entitled: “Zoonotic and Non-zoonotic Parasites of Wild Rodents in Turkman Sahra, Northeastern Iran” published in the Iranian Journal of Parasitology (1). Totally, 25.30% (23/91) of the sampled population were infested with endoparasites which all of them have been introduced as zoonotic parasites. Therefore, more attention should be paid to this item as all endoparasites were zoonotic parasites. They reported that the detected endoparasites comprised of 5 species of helminths and reported finding of the larval stage of *Angiostrongylus* spp. in the lung impression smears of two *Mus musculus* and emphasized it *Angiostrongylus* spp. which they showed it with a figure in the article (1).

However, the methodology was not clearly described to understand how *Angiostrongylus* spp. was differentiated in impression smear of lung. No more criteria including morphology, morphometric measurements or molecular work have been stated, so far. Usually, different criteria are used for such reports (2). Therefore, we have some concerns about the methodological aspects of the study and the conclusions are drawn.

*Angiostongylus* is a zoonotic parasite with different species. Adults inhabit the pulmonary arteries and proper atrium, ventricle and vena cava, bronchioles of the lung or arteries of the caecum and mesentery which depends on the species of the worms. Twenty-one species of angiostrongylus has been recognized presently in natural world (3). Those stand up evidently in rodents, tupaiids, mephitids, mustelids, procyonids, felids, and canids, and aberrantly in a number avian, marsupial and eutherian hosts which include humans. All species bypass first-stage larvae within the feces of the host and all utilize slugs and/or aquatic or terrestrial snails as intermediate hosts. Two species, *A. cantonensis* and *A. costaricensis*, are zoonosis and cause human neurological and abdominal angiostrongyliasis respectively (3). Adults *Angiostrongylus* are filiform in both sexes while their anterior end is tapering. The adult male nematodes are 15.5–23.0 mm long and 0.25–0.35 mm wide, usually transparent and possessing a smoothly rounded head. In their oral cavity, two or three-minute triangular teeth are observed. The esophagus is 0.29–0.35 mm long. The excretory pore located in the anterior part of the worm in the region of the esophageal-intestinal junction. Females are larger, measuring 18.5–33.0 mm in length and 0.28–0.5 mm wide. Their head and esophagus are similar to the male. The ovaries are located in the posterior region. The vulva is a transverse slit located about 0.25 mm from the posterior end. The first-stage larva of *Angiostrongylus* is about 0.27–0.30 mm in length and has a distinct notch on the tail while the second stage larva measures 0.42–0.47 mm. The third stage larva is 0.42–0.49 mm. The fourth-stage larva, found in rats but not intermediate hosts, is 0.85–1.0 mm long and has a more pointed tail than earlier stages (4). Detailed description of each stage used for morphological studies, has been described earlier (5–7). They could be considered during morphological studies. Regarding the morphometric measurements, the details of the third-stage larva of different *Angiostrongylus* spp. including length, maximum width, length of esophagus, etc. (8), used for diagnostic purposes. Moreover, study on morphological aspects of *A. costaricensis* by light and scanning electron microscopy has been investigated which is helpful (9). However, regarding these items, no data has been mentioned (1).

Different routes of human infection including the wide range of paratenic hosts has been discussed which is very important to be considered. Therefore, speaking on such important zoonotic helminths needs deep investigation on its different aspects (10).

For such papers which report the existence of parasites as a new record in the region, it is suggested working on all aspects of diagnostic criteria. Morphology, morphometric measurements on the diagnostic criteria of the worm/s and molecular biological study on the isolated worm/s would be very useful.

Thus, without reliable methods for detection and diagnosis of *Angiostrongylus* spp. in the region, it is difficult to conclude with certainty that the figure presented in the paper as *Angiostrongylus* spp.

Due to lacking such criteria and as the methodology was not clearly described to understand how *Angiostrongylus* spp. was differentiated in impression smear of the lung including the poor quality of the figure; the conclusion of this paper on the existence of *Angiostongylus* spp. in the natural host is questionable. Moreover, epidemiological studies in the mentioned region do not support the finding of the paper on *Angistrongylus* spp.

## References

[1] Gholipoury M Rezai HR Namroodi S Arab Khazaeli F. Zoonotic and Non-zoonotic Parasites of Wild Rodents in Turkman Sahra, Northeastern Iran. Iran J Parasitol. 2016, 11(3):350–357. 28127340 PMC5256051

[2] Tokiwa T Hashimoto T Yabe T First Report of *Angiostrongylus cantonensis* (Nematoda: Angiostrongylidae) Infections in Invasive Rodents from Five Islands of the Ogasawara Archipelago, Japan. PLoS One. 2013, 8(8): e70729. 23950989 10.1371/journal.pone.0070729 PMC3737349

[3] Spratt DM. Species of Angiostrongylus (Nematoda: Metastrongyloidea) in wildlife: A review. Int J Parasitol Parasites Wildl. 2015; 4(2):178–89 25853051 10.1016/j.ijppaw.2015.02.006 PMC4381133

[4] Lv S Zhang Y Liu H-X Invasive snails and an emerging infectious disease: results from the first national survey on *Angiostrongylus cantonensis* in China. PLoS Negl Trop Dis. 2009; 3(2):e368. 19190771 10.1371/journal.pntd.0000368 PMC2631131

[5] Mackerras MJ Sandars DF. The life history of the rat lung-worm, *Angiostrongylus cantonensis* (Chen) (Nematoda: Metastrongylidae). Aust J Zool. 1955; 3(1):1–21.

[6] Lv S Zhang Y Liu H-X Angiostrongylus cantonensis: morphological and behavioral investigation within the freshwater snail Pomacea canaliculata. Parasitol Res. 2009; 104(6):1351–9. 19172296 10.1007/s00436-009-1334-z

[7] Maldonado A Jr Simões R Thiengo S. Angiostrongyliasis in the Americas. In: Lorenzo-Morales J, ed. Zoonosis. Rijeka, Croatia: InTech; 2012:303–320.

[8] Ash LR. Diagnostic morphology of the third-stage larvae of *Angiostrongylus cantonensis*, *Angiostrongylus vasorum, Aelurostrongylus abstrusus*, and *Anafilaroides rostratus* (Nematoda: Metastrongyloidea). J Parasitol. 1970;56(2):249–53. 5445821

[9] Rebello KM Menna-Barreto RF Chagas-Moutinho VA Morphological aspects of Angiostrongylus costaricensis by light and scanning electron microscopy. Acta Trop. 2013, 127(3):191–8. 23685002 10.1016/j.actatropica.2013.05.002

[10] Barratt J Chan D Sandaradura I *Angiostrongylus cantonensis*: a review of its distribution, molecular biology and clinical significance as a human pathogen. Parasitology. 2016; 143(9):1087–118. 27225800 10.1017/S0031182016000652

